# Investigating the Palatability of Lamb and Beef Components Used in the Production of Pet Food for Cats

**DOI:** 10.3390/ani10040558

**Published:** 2020-03-27

**Authors:** Pavinee Watson, David Thomas, Adrian Hoggard, Michael Parker, Nicola Schreurs

**Affiliations:** 1 School of Food and Advanced Technology, Massey University, Palmerston North 4442, New Zealand; M.E.Parker@massey.ac.nz; 2 School of Agriculture and Environment, Massey University, Palmerston North 4442, New Zealand; D.G.Thomas@massey.ac.nz (D.T.); N.M.Schreurs@massey.ac.nz (N.S.); 3 Ziwi Ltd., Mount Maunganui 3116, New Zealand; adrian@ziwipets.com

**Keywords:** feline, offal, by-products, acceptance, preference, diet, ingredients, red meat, organ meats

## Abstract

**Simple Summary:**

This work investigated the palatability of raw meat components commonly used in the production of pet food for cats. The objective was to develop a ranking of components within lamb and beef, as well as to evaluate whether cats show preference for the same component of one species over the other. Cats showed a general preference for liver and kidney, and for lamb over the equivalent beef components. These results may be useful for pet food manufacturers when developing new products or reformulating existing diets with high meat content to improve overall diet palatability.

**Abstract:**

The pet food industry continues to utilise large amounts of inedible meat components from the human food industry. Although used extensively in pet food formulations and as palatants, little is known about the palatability of individual meat components. The objectives of this study were to investigate the palatability of raw meat components commonly used in the production of pet food, using acceptance and preference testing. Those examined were lung, heart, kidney, tripe, liver and mechanically deboned meat (MDM) from lamb and beef. Two-bowl acceptance tests were used to develop an overall ranking of components within each species. Two-bowl preference tests between equivalent beef and lamb components were then used to determine whether a preference was exhibited for one species over the other. For the acceptance of components from lamb and beef, liver was the most palatable within both species, with kidney equivalent to liver when testing lamb components. The MDM was identified as the least palatable component from both species. When examining the preference between equivalent components between species, cats showed preferences for lamb over equivalent beef components, except for heart and liver which showed no difference in intake between the two species. Overall, cats were able to clearly rank the palatability of different components from lamb and beef, as well as between equivalent components from the two species. Selecting highly palatable ingredients whilst still meeting pet food manufacturing guidelines may play a role in improving overall diet palatability and acceptance by cats.

## 1. Introduction

Palatability is an extremely important criteria in the production of pet food and can often determine the success or failure of a product. Palatability is described as the physical and chemical properties of the diet, which are linked with promoting or suppressing feeding behaviour during the pre-absorptive period [1,2]. Rather than being related to an appetite or craving that indicates a want or need, palatability relates to taste pleasure, liking or happiness [3]. Although the primary aim of pet foods is to deliver complete and balanced nutrition, it must also be perceived by pet owners as being “liked” and palatable by their pets and, therefore, worthy of repurchasing. In addition to complete and balanced foods, palatants are also important in the production of pet treats. For these reasons, palatability remains a focal point in pet food research and development.

Palatability studies are commonly used by manufacturers to assess the acceptance of complete diets when developing new products, as well as the preference between diets undergoing reformulation. Palatability plays an important role in food preference in domestic cats [4]. Testing involves the use of multiple animal subjects and is generally repeated over multiple days to eliminate environmental influences [5]. The animals are usually placed in individual testing booths to avoid distraction, social interaction and competition whilst they are given free access to food for a defined time period [6]. By measuring food intake, it is possible to evaluate the acceptance of new diets, and comparative intakes between different diets to observe whether there is a preference for one over the other [2].

From a nutritional perspective, cats are known as prey-driven animals, formally termed obligate carnivores [2]. They require certain nutrients to be present in their diets such as taurine and arachidonic acid and have a higher requirement for other nutrients such as arginine and niacin compared to canine diets [2,3,7,8,9]. Cats can also detect small differences in the composition of food they are offered [9]. Generally, they are drawn to foods with a strong umami (or savoury) flavour, which is often related to a high concentration of amino acids [10,11]. Furthermore, the abundance of amino acid taste receptor units on the tongues of cats may also be associated with meat eating and are likely used to discriminate between meats of different quality [12].

Cats require vital nutrients in their diet, some of which are found to be more abundant in animal tissue. However, little is known about the palatability of individual ingredients. Meat and meat components are used extensively and have been identified as a major contributor to the growth and expansion of the pet food industry [8,13]. It is hypothesised that cats will be able to detect differences in the palatability of selected components used as major ingredients in pet food formulations. The objectives of this study were to determine whether cats showed varying levels of acceptance for different components within lamb and beef and examine their preference for the same component between species when presented raw. This study, therefore, primarily aims to answer the research question “do cats prefer lamb or beef?” With the secondary aim being “do cats rank the palatability of selected components within lamb and beef?”

## 2. Materials and Methods

All animal procedures described were approved by the Massey University Animal Ethics committee (Protocol MUAEC 18/16).

### 2.1. Experimental Design

Using a test panel of eight cats, a series of three experiments were performed to evaluate the acceptance and preference of different components. The first experiment assessed lung, heart, kidney, tripe, mechanically deboned meat (MDM) and liver from lamb. For the second experiment, the same components from cattle (beef) were evaluated. The third experiment compared a single component from beef and lamb (lung, heart, kidney, tripe, MDM or liver) in a series of tests to determine whether there was a species effect on preference. For each of these experiments, 40 measurements for each component (or pairs of components) were obtained over a five-day testing week.

### 2.2. Test Subjects

A designated panel of eight, domestic short-haired cats was used to test the acceptance of six lamb and six beef components, as well as the preference for the same component between species. The cats used in this study were healthy and consisted of four entire females and four castrated male cats aged from 18 months to six years of age (average age of 3.7 ± 0.6).

### 2.3. Raw Materials

Components were collected over a six-month period prior to palatability testing (November 2017 to March 2018) and sourced from MPI-accredited meat processors through a New Zealand premium pet food manufacturer, Ziwi Ltd. (Mount Maunganui, New Zealand). All raw materials arrived in April 2018 and were held frozen at −27° before sample preparation in May 2018, and testing which took place from 28 May until 19 October 2018.

### 2.4. Sample Preparation for Acceptance Testing

For the acceptance tests, the order of evaluation was: lung, heart, kidney, tripe, MDM and liver, firstly with lamb and then beef. All components were cut into 2 × 2 × 2 cm (≈5 g) cubes using a band saw and then separately vacuum packed into 2 kg portions, except for the MDM. The MDM showed signs of crumbling once cut, so it was cut into larger blocks of approximately 250 g before being vacuum packed into 2 kg portions. All prepared bags of sample were refrozen in a −27 °C freezer.

On each day prior to testing, one bag of component was placed in a 7 °C refrigerator to thaw overnight. On the test day, the sample was placed in a 1.00 mm-aperture steel sieve to separate the solid matter from the excess purge. Once separated, all testing bowls were filled and left to stand for two hours to ensure that all samples were at room temperature before palatability testing commenced.

### 2.5. Acceptance Testing Protocol

A two-bowl test was used to determine the total intake and percentage consumption of each lamb and beef component [5,6]. Eight cats were placed in individual testing booths for one hour each day over five days to test each component. They were presented with 100 g of the same component in each bowl, with the exception of liver which was presented at a lower amount to avoid vitamin A toxicity. Liver is known to have a high vitamin A content and a safe maximum daily intake of vitamin A for cats is 333,300 IU/kg of diet [14]. The vitamin A concentration in raw lamb liver and raw beef liver on a wet (as fed) basis is 514,467 IU/kg and 943,967 IU/kg, respectively [15]. As the cats are presented with approximately 300 g canned diet (in line with the allocated amount for cats in the Massey University Feline Unit), this meant that the 300 g of intake was only allowed to provide 99,990 IU of vitamin A.

Because the vitamin A content of the canned diet was unknown we divided the maximum vitamin A concentration in the whole diet (i.e., 333,330 IU/kg) by the estimated vitamin A in the liver (also in IU/kg) to provide the proportion of liver that could be fed in the diet if we assumed the total diet (liver and canned) has 333,300 IU/kg (or 99,990 per 300 g). Therefore, for lamb liver it could comprise 64.8% of the diet equivalent to 194.4 g in 300 g and for beef liver it could comprise 35.3% of the diet equivalent to 105.9 g per 300 g.

As testing was carried out over five days, we wanted to be very cautious with the amount of liver that was fed and ensure there were clearly going to be no welfare issues due to vitamin A toxicity. The calculated amount was divided by three to provide a generous safety margin to cover any variabilities in the estimated versus actual amount of vitamin A contained within the liver and canned diets and any variability in intake of the canned diets on the day the liver was provided.

For each component, 40 measurements were produced. Cats were removed before one hour had elapsed if they had consumed all components from both bowls.

The cats were given the components for testing at 10:00 am each day. Following testing, the cats were fed their usual commercially prepared canned diets that were available until the following morning when they were removed at 8:00 am. This provided a two-hour fast prior to feeding the component for testing. The commercially prepared canned diets fed during non-testing periods include a selection of mixed offal (including all the components tested in our work). These canned products formed part of the range of flavours of food offered to the cats to make up the balance of their daily intake during testing, so no offal we tested was novel to the cats.

### 2.6. Sample Preparation and Preference Testing Protocol

All the frozen blocks of beef and lamb components were prepared in a similar manner as the acceptance testing, with the following amendments. Rather than the 2 kg used for acceptance tests, 1 kg portions of each component were vacuum packed for the preference tests. On each day prior to testing, one bag of beef and one bag of the equivalent lamb component were placed in a 7 °C refrigerator to thaw overnight. During preference testing, the cats were presented with two bowls, one with 100 g beef and the other with 100 g of the equivalent lamb component, with the exception of liver. The positions of the two bowls were alternated each day to remove any possibility of cats showing a positional bias [5].

In order to present equal amounts of beef and lamb liver, the vitamin A content in lamb and beef liver was averaged to give a value of 729,217 IU/kg. Liver could comprise 45.7% of the diet (22.8% lamb and 22.8% beef liver) equivalent to 68.6 g of each per 300 g. This value was divided by three as previously used in Section 2.5. Accordingly, 22.5 of lamb liver and 22.5 g of beef liver were presented to the cats for preference testing.

### 2.7. Data Collection and Statistical Analyses

The weight of the bowl with food or any residual food was recorded before and after it was provided to the cat. Acceptance and preference were determined as both food intake (g) and converted to a percentage consumption (%) to account for the difference in the amount of liver presented to the cats, using the following equations:

(1) Food intake (g)=weight of bowl before (g)−weight of bowl after (g)

(2) $$Percentage\;consumption=\frac{Food\;intake\;\left(g\right)}{Initial\;weight\;of\;offal\;\left(g\right)}\times 100$$

For acceptance testing, Tukey analysis was carried out in Minitab 18 (Minitab Inc., State College, PA, USA) and used to determine statistical differences between the percentage consumption of all the possible pairings of components using a significance level of *p* < 0.05. Grouping from the Tukey analysis was also used to develop a final rank of component acceptance. Interactions between component intake and day of testing, as well as cats and component intake were also analysed using a PROC mixed model in SAS 9.4 (SAS Institute Inc., SAS Campus Drive, Cary, NC, USA).

For preference testing, paired *t*-tests were carried out in Minitab 18 (Minitab Inc., State College, PA, USA) and used to determine whether there were statistical differences between the overall intake of equivalent beef and lamb components for each test.

## 3. Results

### 3.1. Lamb Acceptance Testing

**Figure 1:**
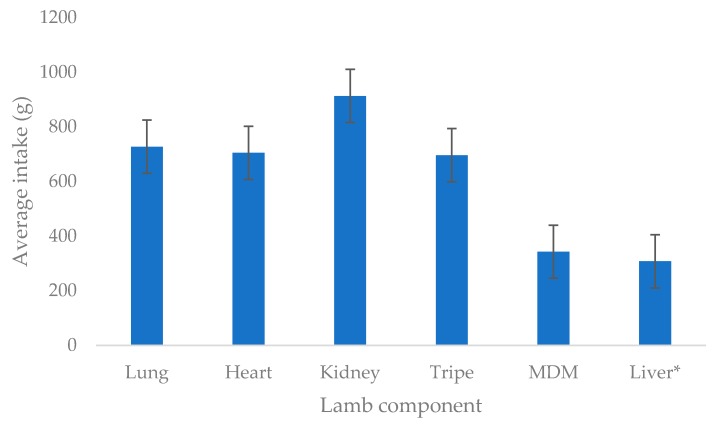
Average intake of the six lamb components out of the possible 1000 g served throughout the week (* maximum possible intake of liver was 325 g compared to 1000 g for the other components).

All cats were offered 1 kg of lamb component each week (200 g a day), except in the case of liver where 325 g was provided weekly (65 g a day). The total intake over the testing period for each component (±SEM) was 727.1 ± 69.9 g for lung, 704.4 ± 73.1 g for heart, 912.5 ± 53.8 g for kidney, 696.0 ± 65.6 g for tripe, 342.8 ± 53.1 g for MDM and 307.8 ± 9.7 g for liver and is given in Figure 1.

For the components that had a maximum possible intake of 1000 g throughout the week, kidney had the highest intake (*p* < 0.05), followed by lung, heart and tripe which showed comparable total intakes (*p* > 0.05) and MDM with the lowest intake (*p* < 0.05). The panel members consistently consumed nearly all the liver presented throughout the week. Within each component, the day of testing had no influence on the intake results (*p* > 0.05). However, intake levels were different for the components tested (*p* < 0.05).

Table 1 shows the percentage consumption of each lamb component for each cat, the average percentage consumption for the cat panel and the final ranking of component acceptance.

Liver was the component most accepted by cats with 94.7% consumption and ranked equally (*p* > 0.05) with kidney at 91.3%. The second ranked (*p* < 0.05) components were lung, heart and tripe at 72.7%, 71.0% and 69.6%, respectively. MDM was least accepted (*p* < 0.05) by cats with a percentage consumption of 34.3% (Table 1; *p* < 0.05).

### 3.2. Beef Acceptance Testing

**Figure 2:**
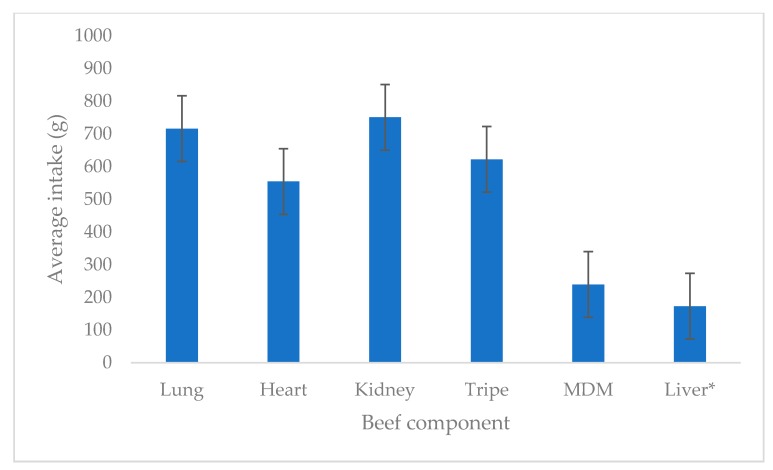
Average intake of the six beef components out of the possible 1000 g served throughout the week (* maximum possible intake of liver was 175 g compared to 1000 g for the other components).

All cats were offered 1 kg of beef component each week (200 g a day), except in the case of liver where 175 g was provided weekly (35 g a day). The total intake over the testing period for each component (±SEM) was 716.0 ± 67.6 g for lung, 554.4 ± 110.9 g for heart, 750.6 ± 106.2 g for kidney, 622.1 ± 100.4 g for tripe, 239.4 ± 40.0 g for MDM and 172.9 ± 0.4 g for liver and is given in Figure 2.

For the components that had a maximum possible intake of 1000 g throughout the week, lung, kidney and tripe, as well as lung, heart and tripe showed comparable total intakes (*p* > 0.05), with MDM showing the lowest intake (*p* < 0.05). The panel members consistently consumed nearly all the liver presented throughout the week. Within each component, the day of testing had no influence on the intake results (*p* > 0.05). However, intake levels were different for the components tested (*p* < 0.05).

Table 2 shows the percentage consumption of each beef component for each cat, the average percentage consumption for the whole cat panel and the final ranking of component acceptance.

Liver was the component most accepted by cats with 98.8% consumption, ahead of all the other beef components (*p* < 0.05). Kidney was the second most palatable ingredient along with lung and tripe (62.2–75.1% consumption; Table 2). Lung and tripe were also ranked equally (*p* < 0.05) with heart below these with 55.4% consumed. However, heart was less palatable than kidney (*p* < 0.05; Table 2). MDM was least accepted by cats with a percentage consumption of 23.9% (*p* < 0.05; Table 2).

### 3.3. Preference between Equivalent Lamb and Beef Components

**Figure 3:**
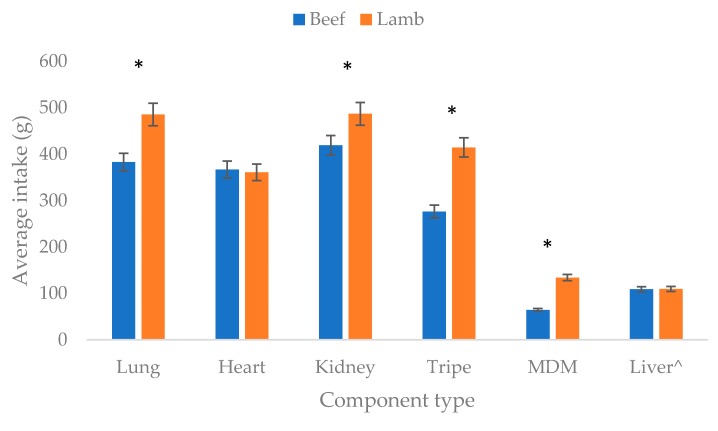
Average intake of equivalent beef and lamb components offered in the two-bowl preference test. (Results with an asterisk indicate a significant difference in intake between equivalent components (*p* < 0.05), ^ maximum possible intake of each liver was 113.5 g compared to 500 g for the other components).

All cats were offered 500 g of each equivalent beef and lamb component each week (100 g a day), except in the case of liver where 113.5 g (22.5 g a day) was presented. The average intakes of all component are given in Figure 3.

In summary, the cats showed a higher intake of lamb lung, kidney, tripe and MDM compared to the beef variety (*p* < 0.05). However, similar intakes of heart and liver were observed between lamb and beef (*p* < 0.05).

## 4. Discussion

This research investigated the palatability of commonly utilised beef and lamb components in pet food. The components were tested by cats in raw form to remove the effects of thermal processing. The objective was to develop a ranking of components within lamb and beef, as well as evaluate whether cats show a preference for the same component of one species over the other.

In the lamb acceptance testing, liver and kidney were identified by the panel of cats as being equally highly palatable (*p* < 0.05) and top ranked. Lung, heart and tripe were also identified as equally palatable (*p* < 0.05) but ranked second, and MDM was ranked the least palatable of all the ingredients with the lowest intake. The results indicated that cats show varying levels of acceptance for different lamb components and highlights that cats can detect small differences in the composition of food they are offered [9], and more specifically, the differences in the palatability of individual ingredients.

In the beef acceptance testing, liver was identified by the panel of cats as being the most palatable of all the beef components (*p* < 0.05). Kidney was the second most palatable ingredient along with lung and tripe (*p* < 0.05). Heart was ranked third also alongside lung and tripe (*p* < 0.05), and MDM was ranked the least palatable of all the ingredients with the lowest intake. The beef results were more complex with four ranks compared to three ranks in lamb, and the overlapping of lung and tripe between rankings two and three, something which did not arise in the lamb acceptance testing. This indicated that, in terms of the four clear rankings, the cats were able to discriminate between the liver, kidney, heart and MDM easily. However, similarities in flavor and aroma made it difficult for the cats to sense differences between kidney, tripe and lung, as well as between tripe, lung and heart, therefore resulting in overlapping levels of acceptance.

Overall, liver was identified as the most palatable ingredient in both lamb and beef at 94.7% and 98.8% consumption, respectively, with lamb kidney also being equally palatable to lamb liver at 91.3% consumption. However, the high vitamin A content that liver possesses often limits its inclusion in pet food, as well as the amount that could be fed relative to the other components presented in this study. Therefore, these results suggest that lamb kidney is a viable highly palatable and safer alternative to liver for high value pet food. Furthermore, it was clearly shown that MDM, an ingredient that is highly available and used extensively in large volumes within pet food formulations, was a poorly palatable component compared to the other lamb and beef components at intakes of 34.3% and 23.9%, respectively. With such a clear decrease in percentage consumption, incorporating more offal components in pet food may be a better option in future high-value pet foods to help increase overall diet palatability.

When examining the preference for equivalent components between lamb and beef, the intakes of lamb lung, kidney, tripe and MDM were preferred over beef (*p* < 0.05). Similar intakes of heart and liver were observed between lamb and beef (*p* < 0.05). As all components in this study were processed within a six-month timeframe, these results could suggest a possible influence of age driving preference for lamb over beef. In New Zealand, lambs are typically 4 to 9 months when processed for meat and components while cull cows (the likely source of beef components) are typically 5 to 8 years of age at slaughter. It is possible that off-flavours may develop as the animals age. It is also very likely that there are a greater number or stronger cross links of connective tissue in older animals [16], which may influence the textural properties and hence palatability, particularly of MDM which showed the lowest intake in general compared to all other components.

Whilst little has been reported on the palatability of meat ingredients used in pet foods, particularly in cats, this study provides an initial evaluation of the palatability of components within a single species and the difference between equivalent components across species.

It is known that the preference for food is often strongly influenced by the food preferences exhibited in their mothers [4]. Exposure to foods during their mother’s pregnancy via amniotic fluid and in early life can also affect a cat’s feeding behaviour [2,4,7]. It has been shown that cats of two ages, as newborns at 9 to 10 weeks, and at 6 months of age, prefer the familiar chemosensory stimulus and avoid the unfamiliar stimulus [17]. As well as chemical stimuli, limited exposure to different foods in early life can result in preference for that flavour, which is referred to as the primacy effect [3]. Subsequently, when pet owners make a range of experiences available, cats generally display the novelty effect, which is the preference for a new food rather than a pet’s accustomed diet [3,9,18].

Although palatability was examined in this study, texture could be examined in the future to determine its effect on palatability. Additionally, this study focuses on evaluating food intake after a defined one-hour period of testing. However, the Massey University Feline Unit does have the capability to examine real-time intakes using load cells and cameras. In future work it will be possible to assess initial selection (choice) and rate of consumption as additional measures of palatability.

In addition, this study uses cats of two sexual populations based on availability. In this case, entire females and castrated males were used. In future studies, it would be worthwhile examining intakes in spayed females. Studies in intact female rats indicated that food consumption was reduced when the effects of estrogen and progesterone were large [19]. It was also revealed that estrogen acts as a reducing factor of eating [20]. For these reasons, it is likely that food intake difference may vary between entire versus spayed females. However, this research was carried out during the period of feline seasonal anoestrus, during winter [21], so it is likely that estrogen and proestrogen levels were low although we did not assess them.

Furthermore, all components were fed raw in this study to eliminate the effects of processing and production of desirable Maillard products or undesirable products, such as the formation of lipid peroxides that can have implications on the palatability of pet food [7,22,23]. It is possible that the outcomes for palatability of raw components described in this study may differ from palatability results for single components that have been processed. However, this study did not attempt to compare raw versus cooked components, but this could be considered in future studies.

## 5. Conclusions

This study investigated the palatability of lamb and beef components used in the production of pet food when fed raw to cats. The results indicated that cats were able to detect differences in palatability of components from lamb and beef, as well as between equivalent components across the two species. In acceptance testing, liver was identified as the most palatable component, with kidney equivalent to it in the lamb acceptance testing. Furthermore, MDM was identified as the least palatable among the tested ingredients. When examining the preference between equivalent components between species, cats showed preferences for lamb over beef, with the exception of heart and liver, for which there was no difference in intake between the two species.

## Figures and Tables

**Table 1:** Weekly percentage consumption results and final ranking of acceptance for the six lamb components.

Cat	Lamb Component Intake (%)					
	Lung	Heart	Kidney	Tripe	Mechanically Deboned Meat (MDM)	Liver
1	63.5	64.8	95.2	72.6	48.8	99.1
2	94.0	59.8	99.0	53.2	35.8	85.8
3	63.0	82.1	85.9	60.6	38.4	98.5
4	36.5	25.4	55.3	36.9	3.3	77.2
5	88.0	80.3	99.1	91.5	21.2	99.4
6	92.0	80.2	96.9	72.2	40.9	99.7
7	81.0	89.9	99.5	81.3	46.5	99.8
8	63.5	85.5	99.1	88.5	39.3	99.1
Average ± SEM	72.7 ± 3.7 ^b^	71.0 ± 4.2 ^b^	91.3 ± 2.9 ^a^	69.6 ± 4.1 ^b^	34.3 ± 4.1 ^c^	94.7 ± 1.9 ^a^

^a,b,c^ Means denoted with different letters differ significantly at *p* ≤ 0.05.

**Table 2:** Weekly percentage consumption results and final ranking of acceptance for the six beef components.

Cat	Beef Component Intake (%)					
	Lung	Heart	Kidney	Tripe	MDM	Liver
1	73.1	23.8	97.9	83.4	38.0	97.7
2	59.0	50.5	38.0	36.3	23.1	98.3
3	56.1	55.4	89.7	78.1	22.1	98.9
4	37.9	18.9	19.8	2.8	0.8	99.4
5	86.2	83.7	79.9	74.4	33.6	98.9
6	89.4	80.4	97.9	64.5	30.7	98.3
7	78.5	45.2	77.8	82.5	23.4	99.4
8	92.6	85.5	99.5	75.7	19.8	99.4
Average ± SEM	71.6 ± 3.8 ^b,c^	55.4 ± 4.8 ^c^	75.1 ± 5.3 ^b^	62.2 ± 4.9 ^b,c^	23.9 ± 2.7 ^d^	98.8 ± 0.3 ^a^

^a,b,c,d^ Means denoted with different letters differ significantly at *p* ≤ 0.05.

## References

[1] National Research Council (2006). Nutrient Requirements of Dogs and Cats.

[2] Aldrich G.C., Koppel K. (2015). Pet food palatability evaluation: A review of standard assay techniques and interpretation of results with a primary focus on limitations. Animals.

[3] Stasiak M. (2002). The development of food preferences in cats: The new direction. Nutr. Neurosci..

[4] Bradshaw J.S. (2006). The evolutionary basis for the feeding behaviour of domestic dogs (*Canis familiaris*) and cats (*Felis catus*). J. Nutr..

[5] Tarttelin M.F. (1997). Gaining and maintaining market share in a competitive environment: Some views on long and short term pet food testing. Proc. Nutr. Soc. N. Z..

[6] Tobie C., Péron F., Larose C. (2015). Assessing food preferences in dogs and cats: A review of the current methods. Animals.

[7] Zaghini G., Biagi G. (2005). Nutritional peculiarities and diet palatability in the cat. Vet. Res. Commun..

[8] Corbin J.E., Pearson A.M., Dutson T.R. (1992). Inedible Meat, Poultry and Fish By-Products in Pet Foods. Inedible Meat by-Products.

[9] Bradshaw J.W.S., Goodwin D., Legrand-Defrétin V., Nott H.M.R. (1996). Food selection by the domestic cat, an obligate carnivore. Comp. Biochem. Physiol..

[10] Alegría-Morán R.A., Guzmán-Pino S.A., Egaña J.I., Sotomayor V., Figueroa J. (2019). Food Preferences in Cats: Effect of Dietary Composition and Intrinsic Variables on Diet Selection. Animals.

[11] Salaun F., Blanchard G., Le Paih L., Roberti F., Niceron C. (2016). Impact of macronutrient composition and palatability in wet diets on food selection in cats. J. Anim. Physiol. Anim. Nutr..

[12] Bradshaw J.W.S. (1991). Sensory and experiential factors in the design of foods for domestic dogs and cats. Proc. Nutr. Soc..

[13] Michel K.E. (2006). Unconventional Diets for Dogs and Cats. Vet. Clin. N. Am. Small Anim. Pract..

[14] Association of American Feed Control Officials (AAFCO) (2019). Official Publication.

[15] Purchas R.W., Wilkinson B.P. (2013). The Concentration of Selected Nutrients in New Zealand Beef and Lamb Cuts and Offal Items: A Report to Beef + Lamb New Zealand Limited.

[16] Hill F. (1966). The Solubility of Intramuscular Collagen in Meat Animals of Various Ages. J. Food Sci..

[17] Hepper P.G., Wells D.L., Millsopp S., Kraehenbuehl K., Lyn S.A., Mauroux O. (2012). Prenatal and Early Sucking Influences on Dietary Preference in Newborn, Weaning, and Young Adult Cats. Chem. Senses.

[18] Church S.C., Allen J.A., Bradshaw J.W.S. (1996). Frequency-dependent Food Selection by Domestic Cats: A Comparative Study. J. Ethol..

[19] Tarttelin M.F., Gorski R.A. (1971). Variations in food and water intake in the normal and acyclic female rat. Physiol. Behav..

[20] Atsushi F., Hiroko H., Hitomi F., Fukuko K., Tatsuo A., Toshiya F. (2015). Sex differences in feeding behavior in rats: The relationship with neuronal activation in the hypothalamus. Front. Neurosci..

[21] Jemmett J.E., Evans J.M. (1977). A survey of sexual behaviour and reproduction of female cats. J. Small Anim. Pract..

[22] Hagen-Plantinga E.A., Orlanes D.F., Bosch G., Hendriks W.H., van der Poel A.F.B. (2017). Retorting conditions affect palatability and physical characteristics of canned cat food. J. Nutr. Sci..

[23] Tamanna N., Mahmood N. (2015). Food processing and maillard reaction products: Effect on human health and nutrition. Int. J. Food Sci..

